# Performance Prediction of Cement Stabilized Soil Incorporating Solid Waste and Propylene Fiber

**DOI:** 10.3390/ma15124250

**Published:** 2022-06-15

**Authors:** Genbao Zhang, Zhiqing Ding, Yufei Wang, Guihai Fu, Yan Wang, Chenfeng Xie, Yu Zhang, Xiangming Zhao, Xinyuan Lu, Xiangyu Wang

**Affiliations:** 1 College of Civil Engineering, Hunan City University, Yiyang 413000, China; genbao@hncu.edu.cn (G.Z.); fuguihai@hncu.edu.cn (G.F.); 2 Institute for Smart City of Chongqing University in Liyang, Chongqing University, Changzhou 213300, China; 22390016@student.uwa.edu.au; 3 School of Design and Built Environment, Curtin University, Perth, WA 6102, Australia; 4 School of Architectural Engineering, Nanjing Institute of Technology, Nanjing 211167, China; wy1823572519@126.com (Y.W.); m17327386130@163.com (X.Z.); luxinyuan6688@163.com (X.L.); 5 Urban and Rural Construction and Investment Group Limited, Putian 351100, China; 13599889911@163.com; 6 General Contracting Company of CCFED, Changsha 410000, China; zy15802629415@126.com

**Keywords:** cement stabilized soil, fiber-reinforced soil, mechanical strength, waste utilization, Back Propagation Neural Network, Random Forest, beetle antennae search

## Abstract

Cement stabilized soil (CSS) yields wide application as a routine cementitious material due to cost-effectiveness. However, the mechanical strength of CSS impedes development. This research assesses the feasible combined enhancement of unconfined compressive strength (UCS) and flexural strength (FS) of construction and demolition (C&D) waste, polypropylene fiber, and sodium sulfate. Moreover, machine learning (ML) techniques including Back Propagation Neural Network (BPNN) and Random Forest (FR) were applied to estimate UCS and FS based on the comprehensive dataset. The laboratory tests were conducted at 7-, 14-, and 28-day curing age, indicating the positive effect of cement, C&D waste, and sodium sulfate. The improvement caused by polypropylene fiber on FS was also evaluated from the 81 experimental results. In addition, the beetle antennae search (BAS) approach and 10-fold cross-validation were employed to automatically tune the hyperparameters, avoiding tedious effort. The consequent correlation coefficients (R) ranged from 0.9295 to 0.9717 for BPNN, and 0.9262 to 0.9877 for RF, respectively, indicating the accuracy and reliability of the prediction. K-Nearest Neighbor (KNN), logistic regression (LR), and multiple linear regression (MLR) were conducted to validate the BPNN and RF algorithms. Furthermore, box and Taylor diagrams proved the BAS-BPNN and BAS-RF as the best-performed model for UCS and FS prediction, respectively. The optimal mixture design was proposed as 30% cement, 20% C&D waste, 4% fiber, and 0.8% sodium sulfate based on the importance score for each variable.

## 1. Introduction

Cement stabilized soil (CSS) is a routine cementitious material that yields wide applications including leakage-stopping, slope reinforcement, and foundation treatments [1]. However, weak strength and large deformation impede its extensive development. Construction and demolition (C&D) waste resolve the imperfection by physical and bonding strength enhancement. C&D waste particles evolve mechanical support in the CSS matrix due to higher hardness, resulting in better unconfined compressive strength (UCS) performance. The CSS mechanical property is further improved by grinding-incineration treated C&D waste, which represents a positive effect on mortar bonding strength [2,3]. Moreover, C&D waste demonstrates stronger enhancement potential under the excitation of saline solution. Previous literature explores ion activation, proving SO_4_^2−^ ions as outstanding catalysts in cement hydration which reduce the initial setting time by 81.1% [4,5]. In addition, Na^+^ provides an alkali situation, leading to compact hydration products. Furthermore, C&D waste endows CSS with corrosion resistance. Sulfate attack remarkably declines the CSS durability as evidenced by structure failure and sulfate heaving. C&D waste mitigates the deterioration by introducing corrosion-resistant particles [6,7]. On the other hand, the incorporation of C&D waste concurrently renders CSS more prone to crack. The non-homogeneous surface forms weaken the interfacial transition zone (ITZ) compared to that between cement, resulting in undesirable mechanical properties [8,9,10,11]. Increasing cement dosage has been proven to be an enhancer, while the specimens conduct brittle destruction [3]. Therefore, the combinatorial strategies that best improve the mechanical performance of CSS have been thoroughly investigated.

Polypropylene fiber yields the widest application range given its potent improvement in mechanical performance and durability. First, the distributed fiber generates random intersection in the specimen, which represents characteristics of preventing crack generation and suppressing brittle destruction [12,13,14]. Mechanical strengths, therefore, enhance significantly as evidenced by the 115% increment of UCS [15,16]. Previous studies have proven likewise promotion with various fibrous materials [17,18]. Second, polypropylene fiber mitigates the sulfate attack. Dobrovolski et al. [19] summarized that fiber bridge entraps more air, where the voids compensate for the formation of the hydrate phase and expansion. However, previous literature has not extensively explored the coupling effect of cement, C&D waste, and polypropylene fiber on CSS performance as theoretically prediction is always feeble against many variables.

Machine learning (ML) models have therefore been proposed as a potential contender to tackle the hindrance [20,21,22]. ML techniques yield the ability of information extraction and pattern generation based on the given dataset and consequently output the predicted value [23,24]. The prediction demonstrates outstanding accuracy with the multi-variable dataset, which renders the ML approach being extensively explored in recent decades [25,26,27]. Moreover, a conventional laboratory test costs a large amount of time and money to achieve accuracy, while ML techniques forecast the results without laborious work and explicit programming. The baseline models including K-Nearest Neighbor (KNN), logistic regression (LR), and multiple linear regression (MLR) are the most widely used ML models owing to their ease of understanding. However, the simplicity impedes their wider application in the cementitious material domain. Specifically, baseline models emerge with insignificant computing power to address the non-linear relationship between multiple variables (treatment, proportion, etc.) and mechanical properties (UCS, FS, etc.) [28]. Complex ML models are therefore introduced to adequately predict the strength. Artificial Neural Network (ANN), Random Forest (RF), Gaussian process regression (GPR), and Support Vector Machines (SVM) are all ML models that yield wide application in engineering material science. Among these, ANN and RF possess imperative potential as standalone and ensemble models, respectively, due to their successful prediction of concrete compressive strength and conductivity [29,30,31,32].

Hyperparameters on the other hand, hinder the more extensive application of ML approaches. ML models represent reliable workability depending on several hyperparameters. Appropriate ML models consume a large amount of time and effort to conduct trial-and-error methods, locating the accurate parameters. Consequently, swarm intelligence (SI)-based metaheuristic algorithms, including firefly algorithm (FA) [33,34] and particle swarm optimization (PSO) [35,36], are proposed to avoid tedious tuning. However, SI concurrently brings undesired computation intensity [37,38]. Therefore, the beetle antennae search (BAS) algorithm is applied to mitigate the problem. BAS demonstrates benefits comprising easy implementation and convergence, yielding the capability of automatically tuning the hyperparameters [39]. The construction of the algorithm originated from the beetle foraging behavior, leading to the order movement of the group and the optimal hyperparameter combination [40,41].

The purpose of this paper is to experimentally investigate the CSS performance enhancement by C&D waste, polypropylene fiber, and sodium sulfate. C&D waste was incorporated by 10%, 20%, and 30% to substitute cement. The dosing level of polypropylene fiber and sodium sulfate were 1%, 2%, 4% and 0.2%, 0.4%, 0.8%, respectively. UCS test, flexural strength (FS) test, and direct shear test were conducted to examine the coupling enhancement on CSS mechanical properties. The Back Propagation Neural Network (BPNN) and FR with BAS algorithm tuning hyperparameters were employed to predict the UCS and FS performance of CSS.

## 2. Experimental Programs

### 2.1. Materials

The soil and C&D waste within this research were sourced from the construction site of Zhushan Road metro station in Nanjing. All particles were pre-dried and ground to sizes less than 5 mm. The physical and mechanical properties of soil are summarized in Table 1. The Portland cement 42.5 was utilized as a stabilizer and cementitious binder. Polypropylene fiber with a length and density of 10 mm and 0.91 g/cm^3^, respectively, was employed to enhance mechanical performance. Table 2 listed the detailed mechanical properties. Moreover, the air gun was applied to refrain fibrous material from agglomeration. Sodium sulfate was used to provide alkali catalysis. 

### 2.2. Mixture Design

The variables in this research were the content of Portland cement, C&D waste, polypropylene fiber, and sodium sulfate. Each dosing level was determined based on its weight ratio to the pre-dried soil. Particularly, Portland cement and C&D waste to soil ratios were defined as 10%, 20%, and 30%. Polypropylene fiber was incorporated, accounting for 1%, 2%, and 4% of the soil weight. The dosing proportions of sodium sulfate were 0.2%, 0.4%, and 0.8%. As for water, the weight ratio was maintained constantly at 80%. The consequent 81 combinations along with 3 control groups (conventional CSS) were cast for experiments.

### 2.3. Mechanical Tests

UCS, FS, and direct shear tests were conducted to estimate the CSS mechanical performance. The procedure for all mechanical tests was prepared strictly in accordance with GB/T50123-1999 [42]. Soil samples for direct shear tests were shaped as 61.8 mm × 20 mm (diameter × height) particularly. The normal stress was applied vertically at $\sigma_{n}=$ 50, 100,150, and 200 kPa to examine the shear strength parameters. The single-doped specimens used for the direct shear test contained C&D waste at 10%, 20%, and 30% content, while the variable dosage design for UCS and FS specimens was consistent with the aforementioned. Cubic (50 mm × 50 mm × 50 mm) and cuboid (40 mm × 40 mm × 160 mm) specimens were cast, respectively, for UCS and FS tests. After vibrating, mortar samples were wrapped in a thin membrane and cured in the standard curing condition (20 ± 2 °C temperature and 95% relative humidity) until tests were conducted at 7-, 14-, and 28-days of the curing period. On the day before the tests, specimens were shifted from the curing chamber and soaked in 24 °C water for 24 h to pattern the humid working situation. YAW-4206 and DY-208JX automatic pressure testers were employed to conduct the UCS and FS tests with a 0.04 MPa/s loading rate. The average data of three replicated specimens after eliminating the error was recorded as the ultimate test result, listed in Appendix A.

### 2.4. Machine Learning Models

#### 2.4.1. Baseline Models

Baseline models including LR, MLR, and KNN were selected, in contrast to BPNN and RF to assess the prediction accuracy. Regression models (LR and MLR) identify the relationship between predictor and output, possessing the benefits of minimum computation and easy implementation. Equations (1) and (2) display the principles of LR and MLR models.

(1) $$\mathrm{Ln}\frac{p}{1-p}=b_{0}+\sum_{k=1}^{n}b_{k}x_{k}$$

(2) $$Y=\beta_{0}+\beta_{1}x_{1}+\beta_{2}x_{2}+\cdots +\beta_{n}x_{n}$$

Within the proposed equations above, *x_k_* and *p* represent independent and dependent variables; *b*_0_ and *b_k_* stand for constant coefficients; *Y* is the predicted strength of CSS; *x_i_* and $\beta_{i}$ (where *i* = 1, 2, 3, …, *n*) denote the considered variables in laboratory test design and regression coefficient, respectively.

KNN algorithm estimates mechanical performance through similitude between inputting values. Specifically, KNN models detect the most similar observations in the dataset and output the average value as the ultimate prediction [43]. The pre-defined function calculates the distance between neighbors with Euclidean distances, assigning all neighbors with the same weight (Equation (3)) [44,45]. KNN models, therefore, possess the superiority of effective prediction among large datasets [29].

(3) $$d\left(i,j\right)=\sqrt{{\left(y_{i1}-y_{j1}\right)}^{2}+{\left(y_{i2}-y_{j2}\right)}^{2}+\cdots +{\left(y_{in}-y_{jn}\right)}^{2}}$$

where *i* and *j* represent the detected points and d is the abbreviation of Euclidean distance.

#### 2.4.2. Back Propagation Neural Network (BPNN)

**Figure 1:**
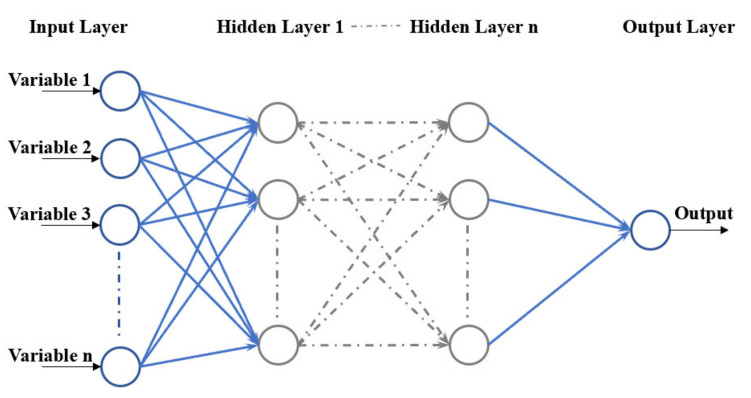
Structure of ANN network.

BPNN, as one type of ANN algorithm trained by the Back Propagation (BP) technique, has been employed to successfully develop the prediction pattern for the mechanical strength of cementitious materials. The ANN model is essentially a neural network, consisting of an input layer, output layer, and hidden layer(s). As illustrated in Figure 1, each neuron yields the ability of a processing unit, merging information from the former layer to transport the combination to the subsequent nodes [46]. The following equation presents the neuron connection between upper and lower layers in the mathematical version.

(4) $$y=\max\left(0,\;\sum_{i}w_{i}x_{i}+b\right)$$

where *y* represents the output value from the lower layer; $w_{i}$ stands for the weight; $x_{i}$ is the received data from the former layer, and *b* denotes the bias between neurons. The neuron loop iterates until the mean squared error (*MSE*) reaches the pre-set value, ending the training process [47].

(5) $$MSE=\frac{1}{n}\sum_{i=1}^{n}{\left(y_{i}-\hat{y_{i}}\right)}^{2}$$

where $y_{i}$ denotes the model prediction, and $\hat{y_{i}}$ is the result estimated by labels.

**Figure 2:**
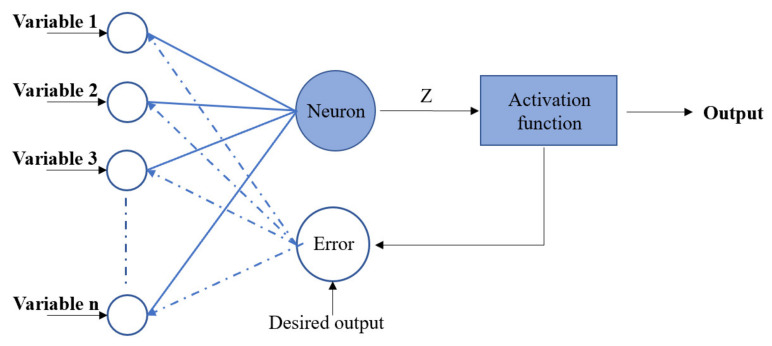
BP procedure.

Figure 2 represents the BP flow, which is the research approach to updating the bias and weights in the neuron network by calculating the difference between the predicted output and the actual strength from the dataset [48,49]. The BP technique endows the BPNN models with sensitivity to hyperparameters which affect the ultimate accuracy.

#### 2.4.3. Random Forest (RF)

**Figure 3:**
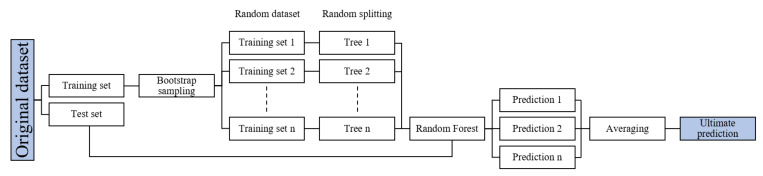
Structure of RF.

As illustrated in Figure 3, RF generates multiple decision trees (RT) in which each RT is built based on a new training set oriented from the bagging and voting method [50]. The bagging method yields characteristics of independently training predictors through bootstrap and aggregation. Bootstrap indicates that RF models allow the duplicate value, which randomly resamples the original dataset by the number of predictors. Each split is built from the random subset selected from the input predictor variables, improving the diversity to achieve accurate estimation. Equation (6) shows the training set as *R_n_* where *X* and *Y*, respectively, denote the input and output vector. The average result of RTs will be output as the ultimate prediction using the aggregation approach [50].

(6) $$R_{n}=\left\{\left(X_{1},Y_{1}\right),\left(X_{2},Y_{2}\right),\ldots ,\left(X_{n},Y_{n}\right)\right\}$$

#### 2.4.4. Beetle Antennae Search (BAS)

The BAS algorithm was proposed from the behavior of beetles, evolving the function to avoid the tedious effort of optimizing hyperparameters manually. The beetles cannot locate the accurate position while looking for food. As a result, beetles move towards the side which receives the greater intensity of odor. Inspired by the principle, the BAS algorithm simulates the goal hyperparameter as the food, rendering the ML models with the capability of automatically tuning [39]. As explained by Equation (7), the first step of BAS is to generate a random vector as the beetle antennae, where *V* indicates the direction and *k* represents the space dimensionality [40].

(7) $$V=rand\left(k,1\right)/\left|\left|rand\left(k,1\right)\right|\right|$$

Secondly, the algorithm determines the antennae coordinate based on the direction vector:

(8) $$X_{l}=X_{i}+D\cdot V$$

(9) $$X_{r}=X_{i}-D\cdot V$$

where $X_{l}$, $X_{r}$, and $X_{i}$, respectively, denote the coordinate of left, and right antennae and their centroid at the *i*th iteration; *D* is the distance between the left and right antennae. The concentration is then compared by the normalized function represented as the following:

(10) $$X_{i+1}=X_{i}+S\cdot normal\left(X_{l}-X_{r}\right)\;f\left(X_{l}\right)<f\left(X_{r}\right)$$

(11) $$X_{i+1}=X_{i}-S\cdot normal\left(X_{l}-X_{r}\right)\;f\left(X_{l}\right)>f\left(X_{r}\right)$$

where *S* represents the length of steps. The comparison iterates 50 times during the model training process to optimize the hyperparameters.

#### 2.4.5. Cross-Validation

**Figure 4:**
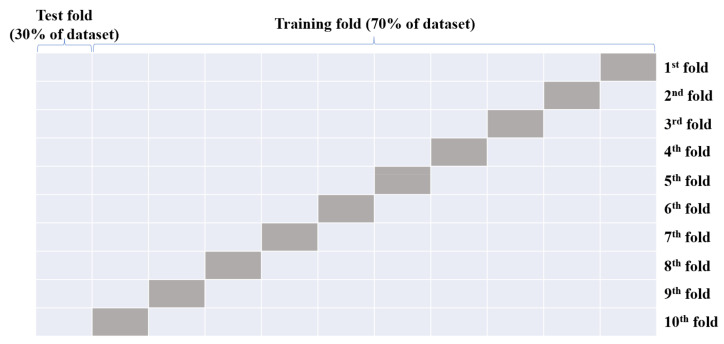
A 10-fold cross validation.

Figure 4 illustrates the 10-fold cross-validation which was applied in this research to mitigate the overfitting during the training and testing stages caused by the finite database. Firstly, the input variable is randomly resampled as training and test set, which, respectively, account for 70% and 30% of the original dataset. Then, the training set is separated into 10 equal folds. The 90% of folds yield the function of training the ML models, and the last fold validates the prediction performance by calculating the root means square error (*RMSE*) [51]. The 10 folds will take turns to be the validation fold. Specifically, in each cross-validation, the BAS algorithm is used to optimize the hyperparameters within 50 iterations. In each iteration, the *RMSE* is calculated for hyperparameter adjustment. Finally, the ML model with the minimum *RMSE* will be saved in each cross-validation (a total of 10 models). By comparing the *RMSE* values from each fold, the ML model with the lowest *RMSE* value and optimal hyperparameters will be chosen as the final ML model.

#### 2.4.6. Performance Evaluation

Two evaluation indicators are applied in this study, aiming to estimate the precision of the baseline, BPNN, and RF models: correlation coefficient (*R*) and *RMSE*. The indexes are defined by the following equations:

(12) $$R=\frac{\sum_{i=1}^{n}(y_{i}^{*}-\bar{y^{*}})\left(y_{i}-\bar{y}\right)}{\sqrt{\sum_{i=1}^{n}{\left(y_{i}^{*}-\bar{y^{*}}\right)}^{2}}\sqrt{\sum_{i=1}^{n}{\left(y_{i}^{*}-\bar{y}\right)}^{2}}}$$

(13) $$RMSE=\sqrt{\frac{1}{n}\sum_{i=1}^{n}{\left(y_{i}^{*}-y_{i}\right)}^{2}}$$

where *n* represents the quantity of data groups; $y_{i}^{*}$ and $y_{i}$, respectively, denote the estimated and actual output; $\bar{y^{*}}$ and $\bar{y}$ are the mean values of $y_{i}^{*}$ and $y_{i}$.

## 3. Results and Discussion

### 3.1. Effect of Portland Cement

**Figure 5:**
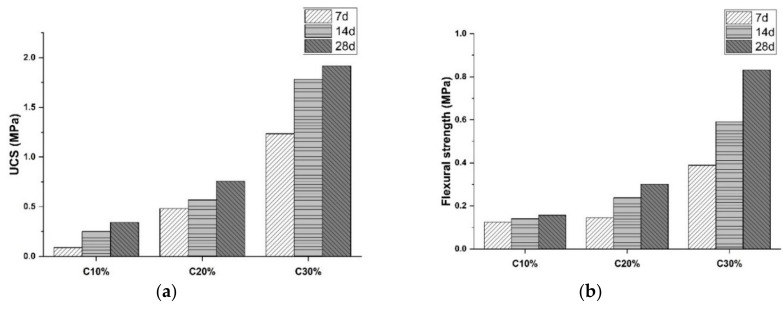
Mechanical results of control groups: (**a**) UCS test, (**b**) FS test (Note: C represents Portland cement).

**Figure 6:**
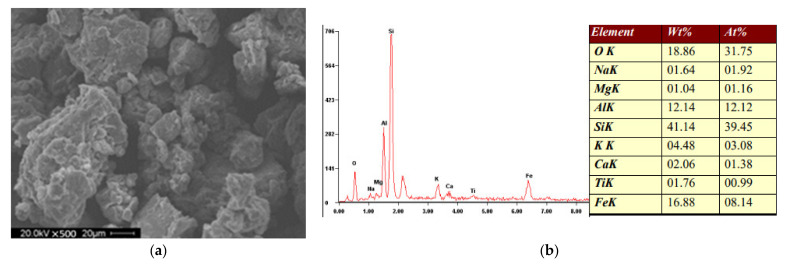
Result of (**a**) SEM, (**b**) EDX.

The UCS and FS test results for control groups are illustrated in Figure 5. It is notable in Figure 5a that the CSS compressive strength increased with the curing age as evidenced by the observed increment up to 185.73% for 7- to 14-day curing time. This is mainly ascribed to cement hydration, which has been proven in Figure 6. Subfigures a and b illustrate the hardened sample photo taken by scanning electron microscope (SEM) and the energy-dispersive X-ray spectroscopy (EDX). The silicon content reached 41.14%, which was higher than that of conventional soil [8]. The results indicate the presence of the hydrate phase like calcium silicate hydrate (C-S-H). These products render strengthened bonding between soil particles and cement. The hydration slowed down to maintain a steady rate during the late curing age (after 14-day), as reflected by the declining increasement ranging from 7.45% to 36.18% in Figure 5. Meanwhile, the UCS test results increased with Portland cement content due to the same reason. The maximum increment during all curing ages is 450.34%, indicating that CaSiO_3_ and C-S-H significantly promote the CSS compressive strength. Specifically, the colloidal hydrate products filled the porosity, contributing to mitigating entrapped air voids. Figure 5b represents a similar trend as FS results at 28-day increased 91.19% and 176.91%, respectively, when cement content rose 10%.

**Figure 7:**
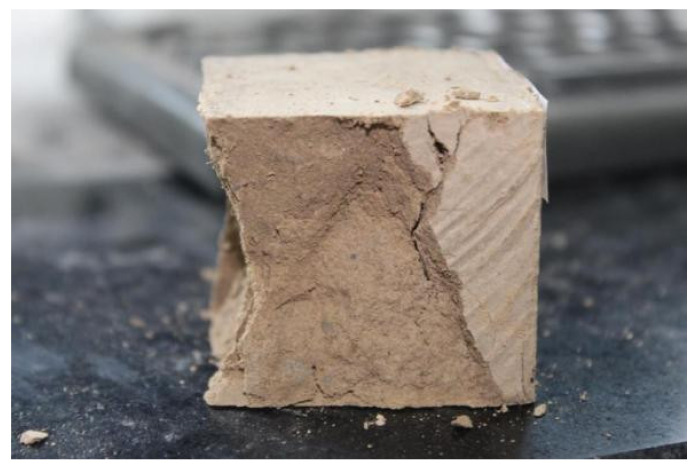
Fracture surface of conventional CSS sample.

On the other hand, increasing Portland cement dosing level causes an undesired alkali–silica reaction (ASR). It is shown in Figure 7 that brittle destruction occurred in control groups, leading to the evident fracture surface. Portland cement introduces brittleness along with compressive strength enhancement, impairing sample deformation to the external force. The conclusion can be demonstrated by the surrounding debris in Figure 7.

### 3.2. Effect of C&D Waste

**Figure 8:**
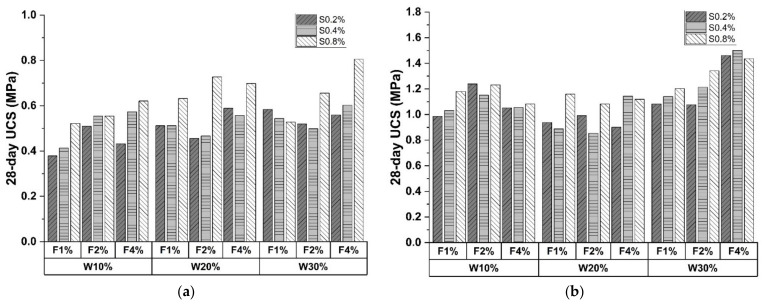

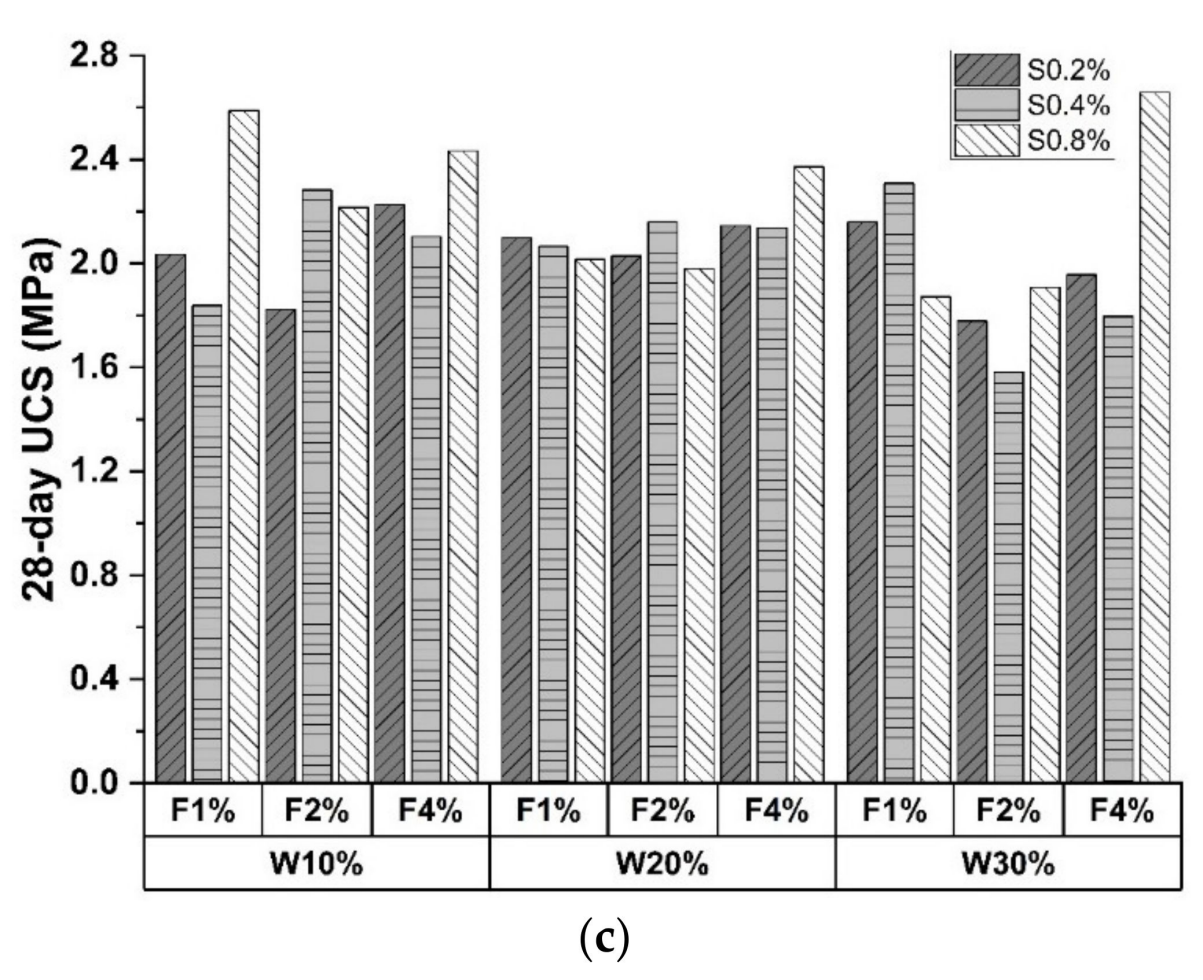
A 28-day UCS of CSS specimens with (**a**) 10% cement; (**b**) 20% cement; (**c**) 30% cement (Note: W represents C&D waste; S represents sodium sulfate; F represents polypropylene fiber).

The 28-day UCS results are shown in Figure 8, with subfigures separated based on Portland cement content. The maximum strength for the three dosing levels as illustrated in the figures were 0.8048 MPa, 1.5008 Mpa, and 2.6572 Mpa, respectively. All the results were higher than those of the control groups, indicating mechanical performance increases with C&D waste incorporation. This is mainly ascribed to the old mortar attached to C&D waste particles which participate in cement hydration [52,53]. The consequent ITZ represents bonding strength that anchors the stiff C&D particles in the matrix to support and prevent soil collapse. Additionally, calcium hydroxide (CH) formed during the hydration process accelerates the hydration process of old mortar, which will generate more calcium silicate hydrates (C-S-H) to promote sample strength [5].

**Figure 9:**
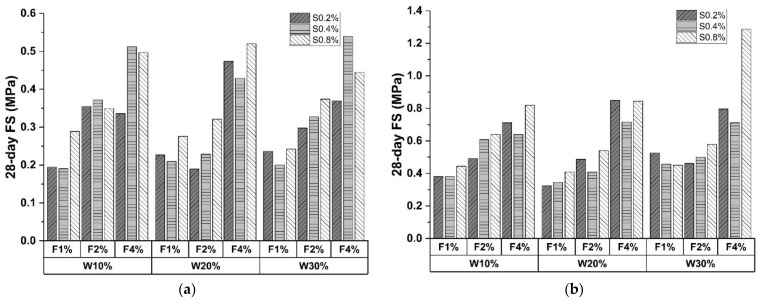

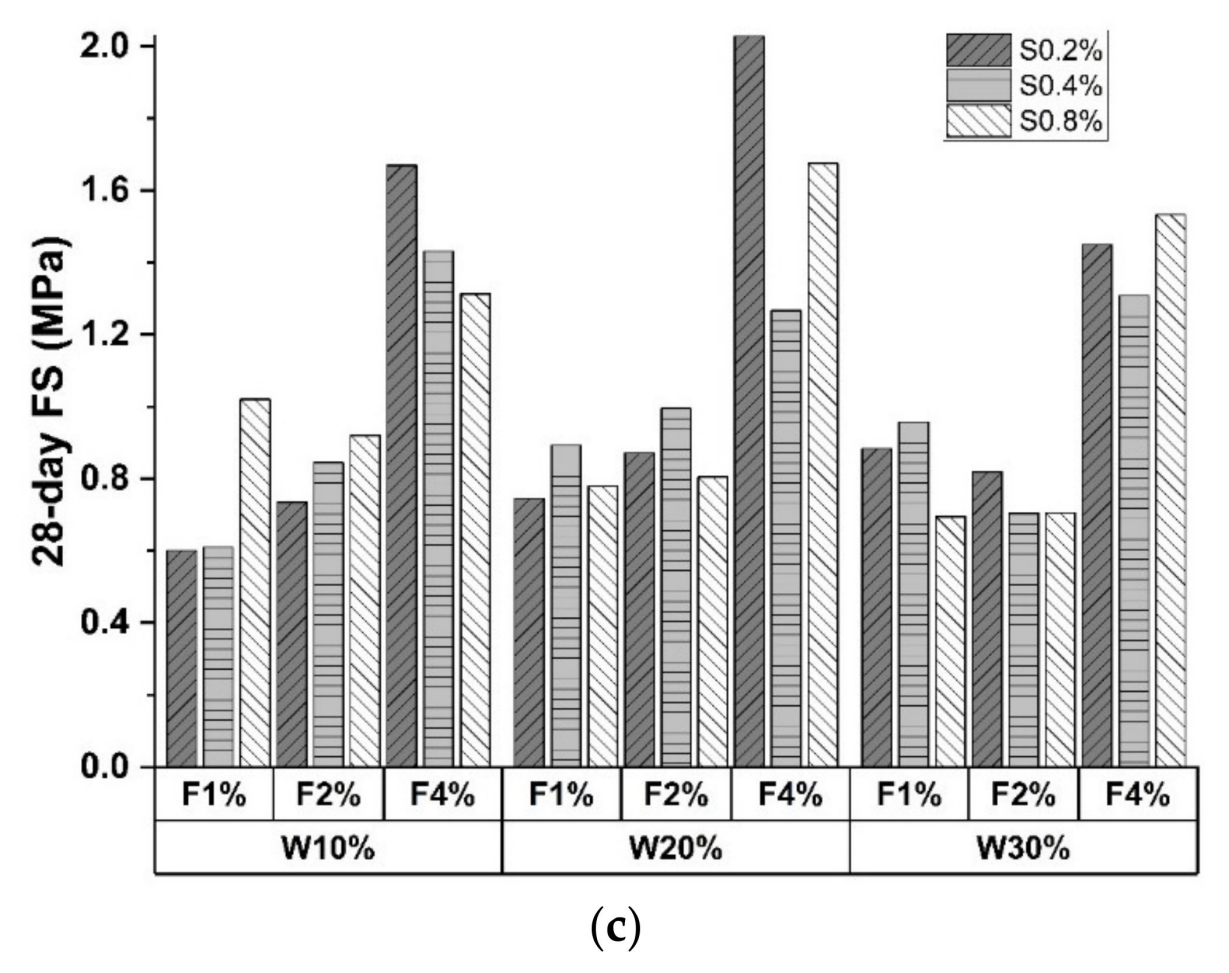
A 28-day FS of CSS specimens with (**a**) 10% cement; (**b**) 20% cement; (**c**) 30% cement (Note: W represents C&D waste; S represents sodium sulfate; F represents polypropylene fiber).

Figure 9 shows the FS results at the 28-day curing age, indicating that C&D waste demonstrates a positive effect on FS performance. As shown in Figure 9b,c, C&D waste displayed better FS enhancement when cement content was high. The maximum improvements reached 56.83% and 57.2%, respectively, while the data in Figure 9a was 21.62%. This phenomenon can be explained as the increase in cement content enhances the degree of hydration of old mortar attached to the C&D waste surface. On the other hand, enhancement became insignificant when C&D waste content was high (30%). This is mainly due to superabundant large particles introduced into the matrix, resulting in porosity and entrapped air.

**Figure 10:**
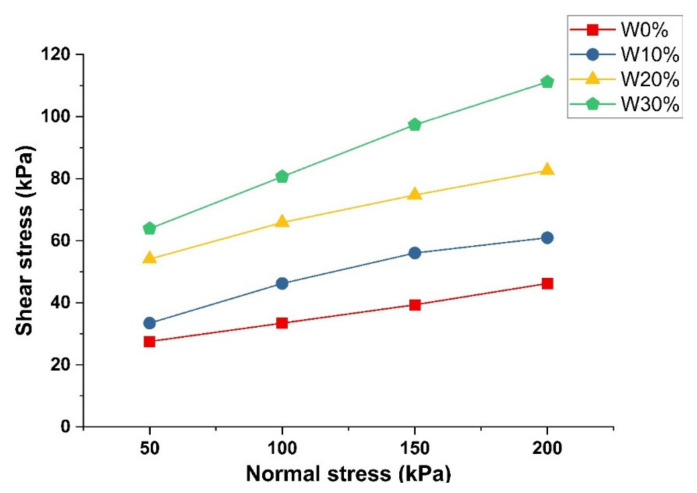
DS test result (Note: W represents C&D waste).

Direct shear tests were also conducted due to their cost-effectiveness and convenient operation. The relationship between normal stress (σ) and shear stress (τ) with various C&D waste proportions is illustrated in Figure 10. The inclusion of C&D waste significantly enhances the shear performance as proven by the increasing cohesion © and material angle of friction (φ). Moreover, the average increments were 33.52%, 43.28%, and 26.34% for each 10% increase in C&D waste dosage, indicating that 20% C&D waste content demonstrated the best-improving effect.

### 3.3. Effect of Polypropylene Fiber

**Figure 11:**
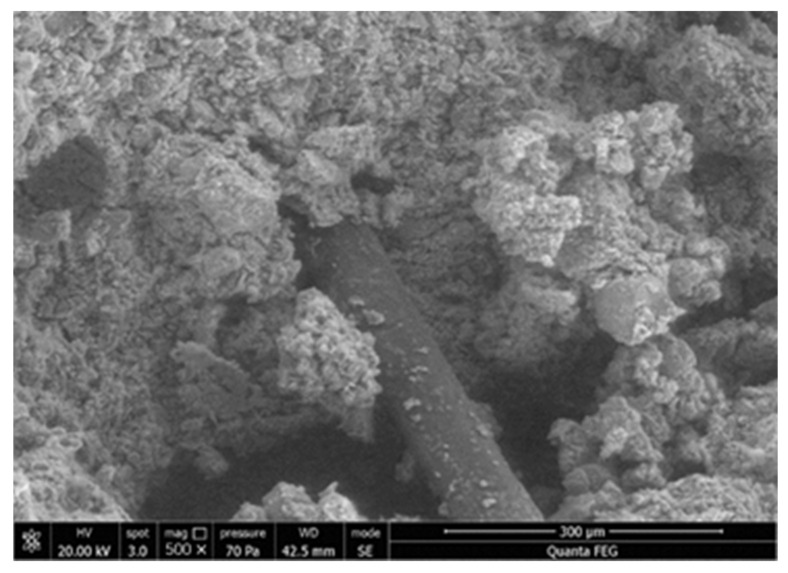
Microstructure of UCS fracture surface.

In Figure 8 and Figure 9, UCS and FS increased with polypropylene fiber proportion, demonstrating the positive effect. The enhancement function on UCS can be attributed to the higher particle friction provided by fiber than that inside the matrix [54]. Specifically, a rougher fiber surface prevents particle displacement, which impedes the generation of microscopic cracks. However, fiber incorporation introduces undesired porosity and compactness descending, leading to a strength reduction of 31.52% in Figure 8c. Figure 11 shows an electron microscope image of the sample fracture surface after the UCS test. Several porosities exist on the fiber periphery. These entrapped air voids caused by fiber agglomeration remarkably weaken the fiber enhancement on compressive strength [55].

Compared with UCS tests, fibrous material promoted FS results more evidently. As depicted in Figure 9a, the FS of CSS specimens increased up to 82.33% and 150.31% when the fiber content doubled. A similar trend can be observed in Figure 9b,c. The dominant enhancement of FS can be attributed to the bridging effect. The randomly distributed fibers demonstrate the outstanding inhibitory function on crack generation. In addition, polypropylene fiber will be pulled out of the fracture surface when the failure occurs, endowing the relict with certain flexural resistance. However, the bridging effective can be hindered by cement inclusion. The peak FS was recorded at 30% C&D waste dosed group when cement content was low (10% and 20%), whereas the 30% cement specimens reached the maximum at 20% C&D waste proportion. This is mainly ascribed to the excessive cement hydration that impeded the fibrous bridge formation.

### 3.4. Effect of Sodium Sulfate

The influence of sodium sulfate can be analyzed in Figure 8 and Figure 9. UCS and FS results share a similar trend that mechanical performances strengthen with sodium sulfate proportion. The average increment of UCS and FS from 0.2% to 0.8% sodium sulfate were 16.94% and 16.29%, with the maximum increasement recorded as 59.61% and 69.96%, respectively. This is attributed to the reaction between sulfate ion and liquid phase (AlO^2−^, Ca^2+^, etc.). The main product is ettringite (AFt phase), revealing enhancing characteristics on early-age strength [56]. Moreover, metal ions (Na^+−^, Ca^2+^, etc.) demonstrate dominant effectiveness in improving alkalinity, which yields function of the reaction rate catalyzation along with SiO_2_ and Al_2_O_3_ dissolution. However, samples incorporated with 0.4% sodium sulfate failed to follow the positive trend. The UCSs of the sample changed irregularly, as evidenced by the value fluctuating from −14% to 32.59% compared to 0.2% sodium sulfate. A similar phenomenon was also observed in Figure 9. The error source is probably ascribed to human error and material composition deviation.

Furthermore, based on Appendix A, the UCS enhancing rate varies from each curing age, as evidenced by the average increment of 15.41% and 30.49% for early (7-day to 14-day) and late (14-day to 28-day) curing times. The principle can be explained by Equations (14) and (15) [57]. Sulfate ions modified the conventional hydration reaction, resulting in the formation of ettringite which promotes the CSS mechanical performance. However, C-S-H exhibits the capability of absorbing sulfate in the early curing stage and releasing it during the later period, leading to rapid strength promotion from 14- to 28-day [58,59,60].

(14) $$C_{3}A+2{\mathrm{CSH}}_{2}+26H\to C_{6}{\mathrm{AS}}_{3}H_{32}$$

(15) $$2C_{3}A+C_{6}{\mathrm{AS}}_{3}H_{32}+4H\to 3C_{4}{\mathrm{ASH}}_{12}$$

## 4. Machine Learning Predicted Results

### 4.1. Prediction for UCS Performance

#### 4.1.1. Hyperparameter Tuning

In total, 252 data (84 groups of experimental results) constituted the database, which reached the requirement of the database size for the traditional machine learning task. During the machine learning process, the contents of cement, C&D waste, fiber, and sulfate, and the curing age were set as features. The outputs were UCS and FS. 

**Figure 12:**
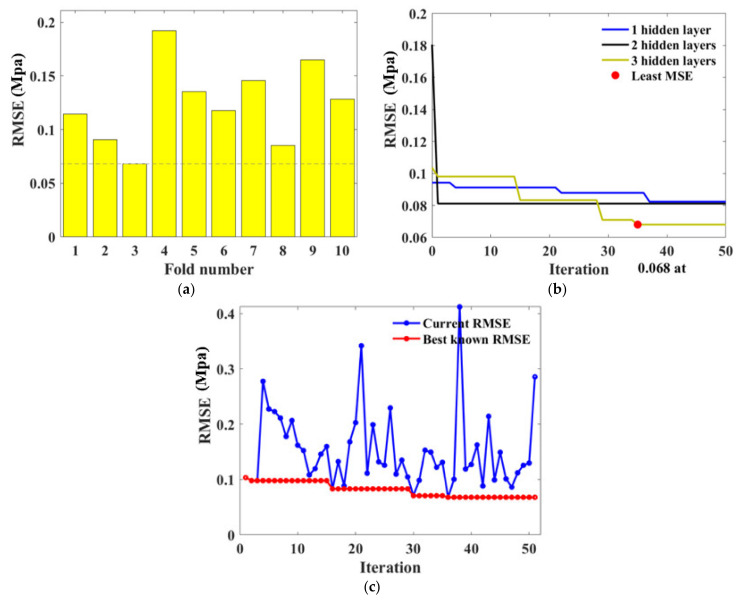
Hyperparameter tuning for BPNN: (**a**) RSME values obtained in 10 validation folds; (**b**) RSME convergency with various numbers of hidden layers; (**c**) Iteration conducted at the 3rd fold.

For BPNN models, hyperparameters that needed to be determined include the number of neurons and layers. BAS and 10-fold CV detected the optimal hyperparameters through iteration as illustrated in Figure 12. It is evident in Figure 12a,b that the third fold and BPNN network with three hidden layers obtained the lowest *RMSE* value. Figure 12c represents the BAS algorithm conducted in fold 3, indicating the *RMSE* value reduced with the iteration and the tuned hyperparameter was gained at the 36th iteration. The consequent BPNN hyperparameters were therefore determined as numHiddenLayers=3, with numNeuronsInEachLayers=3,11,4, respectively.

**Figure 13:**
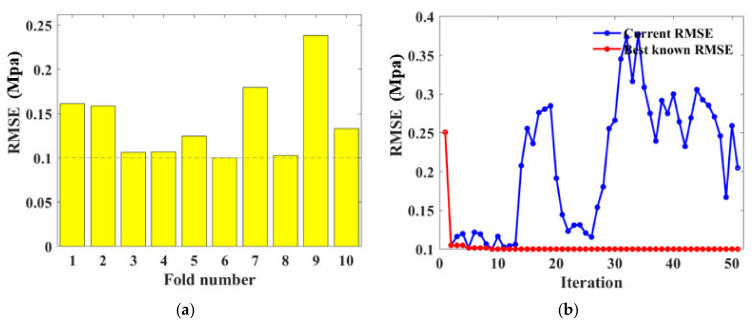
Hyperparameter tuning for RF: (**a**) RSME values obtained in 10 validation folds; (**b**) Iteration conducted at the 6th fold.

During the modeling setting, the amounts of trees (ntree) and the minimum number of leaves (minNumleaf) are fundamental parameters that needed to be adjusted for the RF algorithm. In this research, they were detected from the procedure as shown in Figure 13. It is noted that the *RMSE* value is basically convergent within 50 iterations for the traditional machine learning task. From Figure 12b,c and Figure 13b, the *RMSE* value’s reduction can be clearly observed within the first 10–30 iterations and maintains the minimum value after 30 iterations, illustrating that the *RMSE* reaches the local minimum. Specifically, the minimum *RMSE* value was obtained at the 6th fold as 0.1015 which dropped significantly with iteration progress, demonstrating the obtainment of desired hyperparameters as numTree=88, minNumleaf=1.

#### 4.1.2. Performance of BAS-BPNN and BAS-RF for UCS

**Figure 14:**
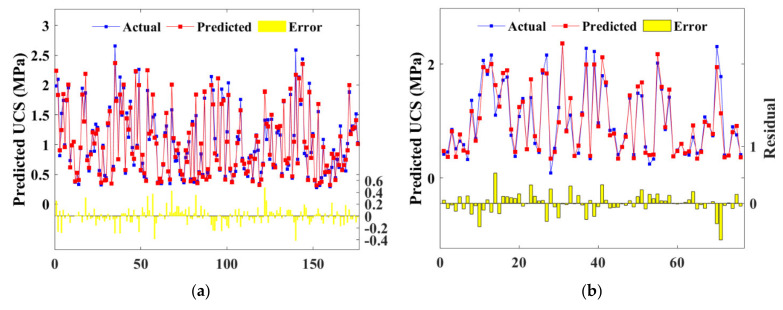
Scatter plot of predicted and actual UCS of BPNN model: (**a**) training set; (**b**) test set.

**Figure 15:**
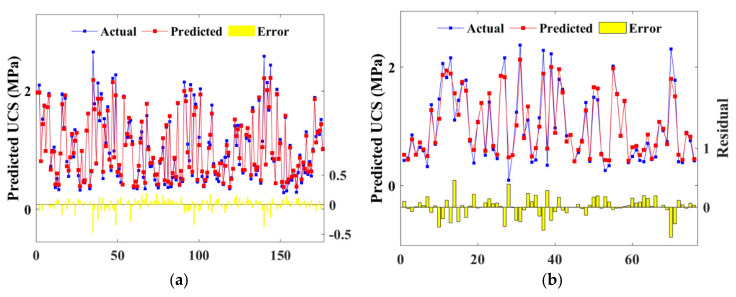
Scatter plot of predicted and actual UCS of RF model: (**a**) training set; (**b**) test set.

Figure 14 and Figure 15 show the prediction performance of BAS-BPNN and BAS-RF, with subfigures (a) and (b) depicting the training and test sets, respectively. The yellow columns denoting the error of prediction were minor, so the consequent conclusion can be drawn that both BPNN and FR estimate the CSS strength accurately.

**Figure 16:**
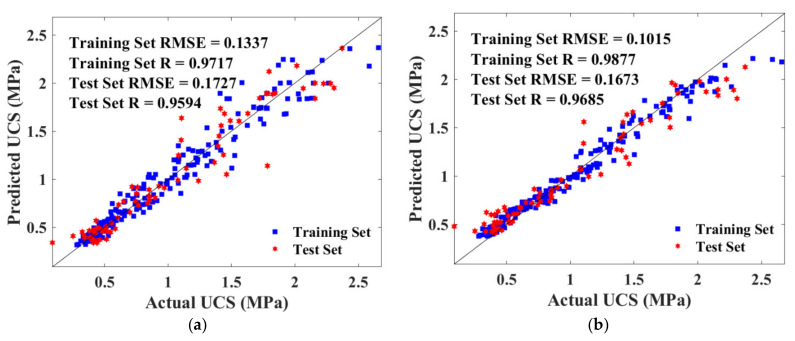
Scatter plot of predicted and actual UCS of training and test sets: (**a**) BAS-BPNN model; (**b**) BAS-RF model.

Additionally, prediction and actual results formed a great correlation as evidenced by the *R* value in Figure 16. As depicted in Figure 16a, the correlation coefficient (R) for the BPNN algorithm were 0.9717 and 0.9594 for the training and test set, respectively, which were both lower than that for the RF algorithm (0.9877 and 0.9685). Therefore, BPNN and RF simultaneously provided reliable predictions, whereas RF yielded enhanced accuracy. Moreover, a similar *R* value indicated that there was no overfitting problem in both algorithms.

#### 4.1.3. Comparison of BPNN. RF, LR, MLR, and KNN

**Figure 17:**
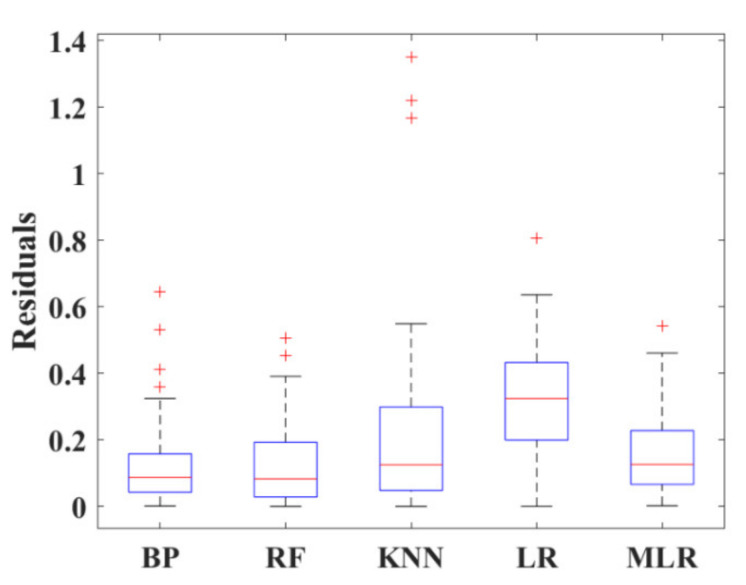
Box diagram of 5 models.

Figure 17 is the box diagram representing the various between the actual strength and prediction. The boxes in the figure indicate the interquartile range for each model by the height between the upper and lower borders. It can be observed that BPNN yielded the best accuracy and shared the lowest median (the red line within the box) with the RF algorithm. Four outliers (read+) were defected in BPNN, which was more than that of other models. However, as the interquartile range and median affect the accuracy more significantly, BPNN and RF demonstrated relatively similar reliability among all five algorithms.

**Figure 18:**
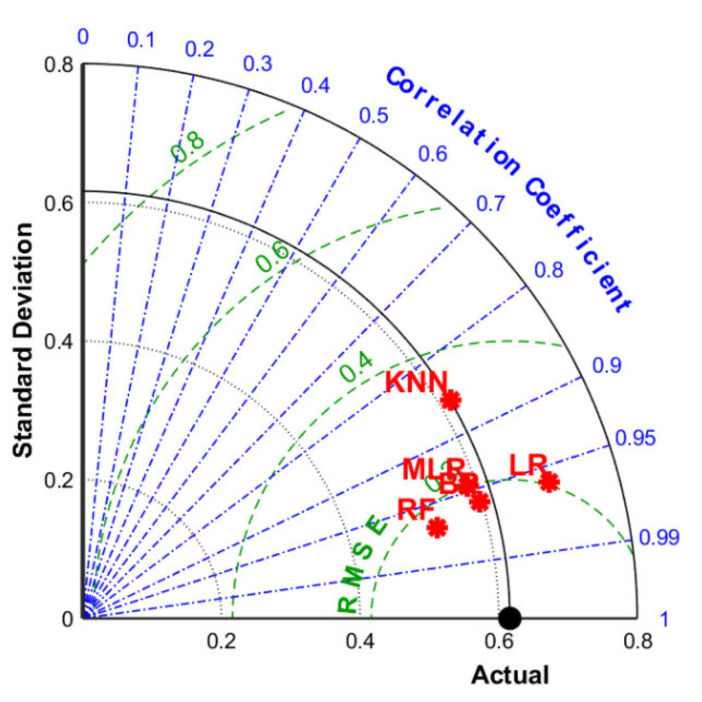
Taylor diagram of 5 models.

The Taylor diagram was also applied to evaluate the model performance through three assessment criteria including *R*, *RMSE*, and standard deviation, as shown in Figure 18. The dot denoted for RF was the nearest to the actual point with the minimum standard deviation, maximum *R*, and minor *RMSE*. Table 3 listed the specific value of *R* and *RMSE* for each algorithm, proving RF as the best-performed model in UCS prediction.

### 4.2. Prediction for FS Performance

#### 4.2.1. Hyperparameter Tuning

**Figure 19:**
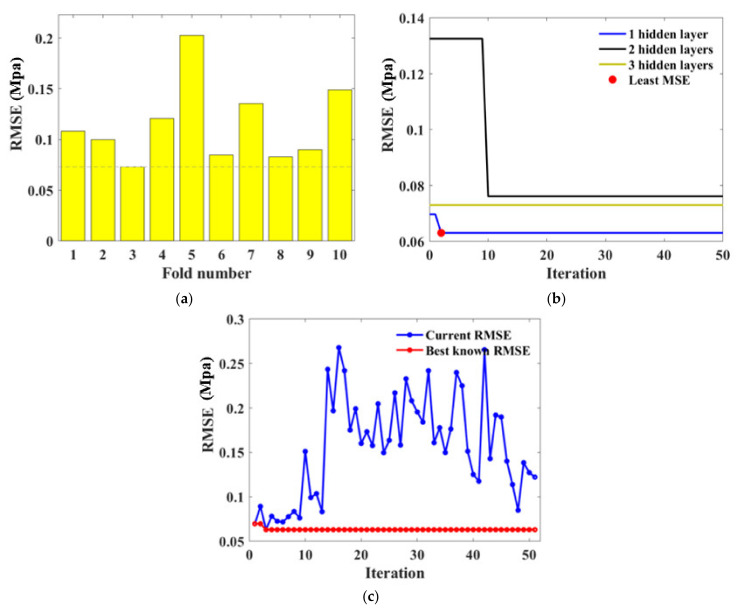
Hyperparameter tuning for BPNN: (**a**) RSME values obtained in 10 validation folds; (**b**) RSME convergency with various numbers of hidden layers; (**c**) Iteration conducted at the 3rd fold.

A similar procedure with UCS estimation was applied to optimize hyperparameters for FS prediction. The 3rd fold outputs the minimum *RMSE* value during the CV process as shown in Figure 19a. Moreover, the numHiddenLayers was examined as 1 because the *RMSE* reduced remarkably and reached the minimum when the iteration was processed for three times. The phenomenon can be ascribed to the effectiveness of BAS on hyperparameter tuning. The other desirable hyperparameter numNeuronsInEachLayers defected as 1.

**Figure 20:**
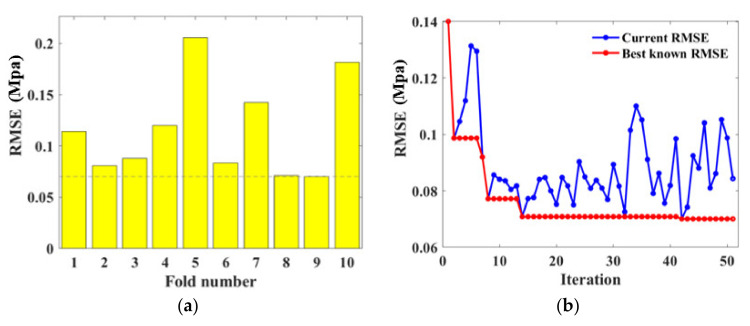
Hyperparameter tuning for RF: (**a**) RSME values obtained in 10 validation folds; (**b**) Iteration conducted at the 9th fold.

For the RF algorithm, the 9th fold had the minor *RMSE* as evidenced by Figure 20a. The hyperparameters optimized by this iteration were therefore applied to predict the FS performance. Figure 20b shows the *RMSE* scatter plot, indicating the decline of *RMSE* value until it maintained a minimum at the 41st repeat. The final tuned hyperparameters were numTree=29, minNumleaf=1.

#### 4.2.2. Performance of BAS-BPNN and BAS-RF for FS

**Figure 21:**
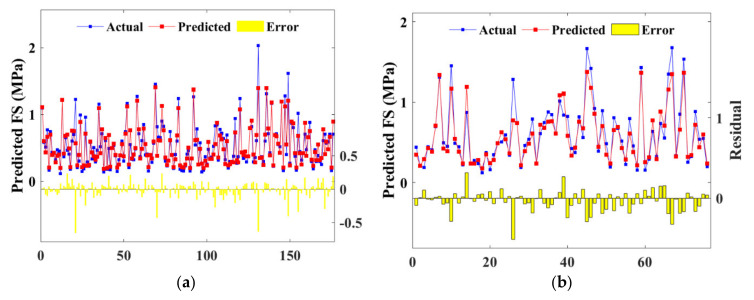
Scatter plot of predicted and actual FS of BPNN model: (**a**) training set; (**b**) test set.

**Figure 22:**
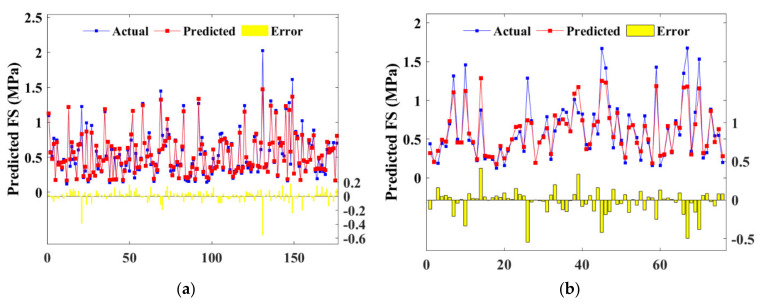
Scatter plot of predicted and actual FS of RF model: (**a**) training set; (**b**) test set.

After being automatically tuned in the 70% training set, hyperparameters were applied in the 30% test set to predict the FS property of CSS. Figure 21 and Figure 22 present the scatter plot of FS prediction from BPNN and RF models with the actual strength of the training and test set, respectively. It is noted from the figures that the prediction and the actual results fitted well, as the contract ratio of red and blue lines were relatively high. Furthermore, error bars located on the horizontal line proved that BPNN and RF algorithms demonstrated similar accuracy in FS prediction.

**Figure 23:**
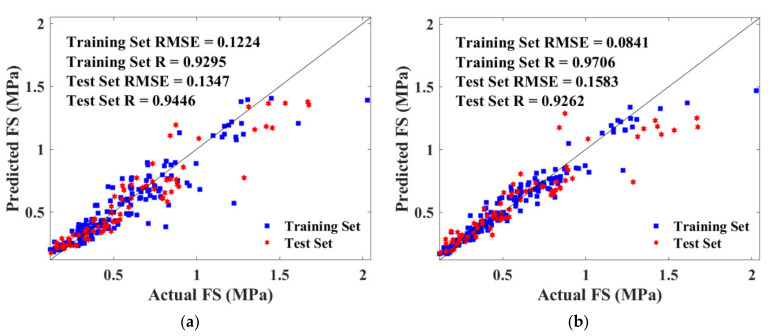
Scatter plot of predicted and actual FS of training and test sets: (**a**) BAS-BPNN model; (**b**) BAS-FR model.

Detailed *RMSE* and *R* value are illustrated in Figure 23, where subfigure (a) depicts the BPNN model, and (b) depicts the RF model. The *RMSE* values were ranging from 0.0841 to 0.1583, indicating that BPNN and RF models estimated the strength accurately. The training set of the RF algorithm defected the hyperparameters with the highest *R* and the lowest *RMSE*. However, the test set output the worst-performed value, which manifested that the RF model had a higher risk of overfitting compared with BPNN.

#### 4.2.3. Comparison of BPNN. RF, LR, MLR, and KNN

**Figure 24:**
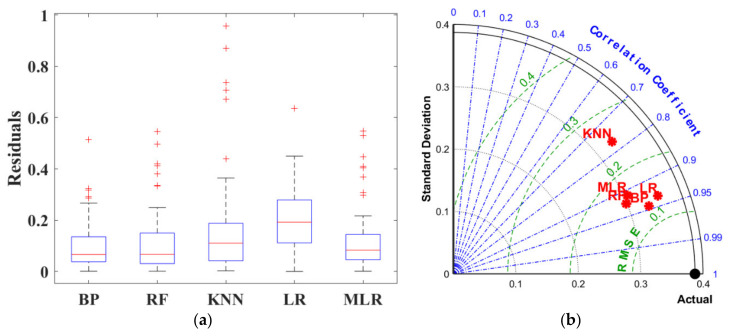
Prediction evaluation: (**a**) box diagram; (**b**) Taylor diagram.

For FS prediction, MLR demonstrated high accuracy as evidenced by the condensed interquartile range in Figure 24a. However, BPNN exhibited better-integrated reliability because of fewer outliers and the lower median value. Figure 24b integrated *R*, *RMSE* and standard deviation into polar coordinates, obtaining the same conclusion owing to the closest distance between BPNN and the actual FS result. In addition, based on evaluation standards listed in Table 4, BAS-BPNN was also considered the most effective algorithm due to the least error and best degree of fitting.

## 5. Optimal Mixture Design

**Figure 25:**
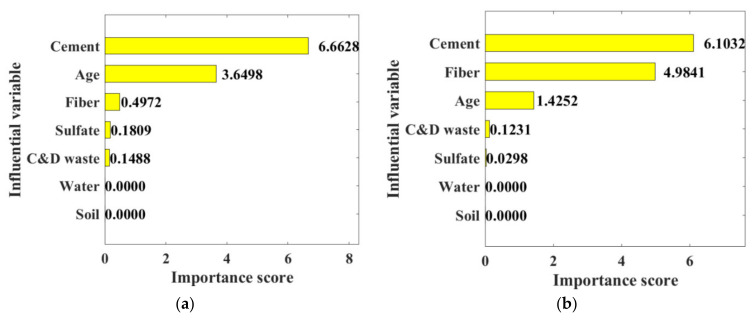
Importance score of influential variables: (**a**) UCS; (**b**) FS.

The RF algorithm defined the effect factor of each variable as depicted in Figure 25, which contributed to proposing the optimum mixture design. The consequent importance score for each content was similar to that obtained from laboratory tests. Water and soil proportion exhibited no influence on the mechanical property owing to the constant dosing level in all specimens. C&D waste and sodium sulfate had a similar introduction effect. It is noted that cement content, curing age, and fiber content yielded the best effectiveness on CSS mechanical strength. Combined with the UCS and FS results listed in Appendix A, specimens prepared with 30% cement, 20% C&D waste, 4% polypropylene fiber, and 0.8% sodium sulfate were considered the best performed. The conclusion can be attributed to the high ranking of the 28-day UCS and FS performance among all mixture designs.

## 6. Conclusions

In this research, the inclusion effects of Portland cement, construction and demolition (C&D) waste, polypropylene fiber, and sodium sulfate on the mechanical properties were assessed through laboratory tests. Moreover, machine learning (ML) techniques were applied based on the 84 experimental results, predicting the unconfined compressive strength (UCS) and flexural strength (FS) of cement stabilized soil (CSS). Primary conclusions are drawn as follows:

(1) Portland cement demonstrates outstanding enhancement of mechanical strengths through cement hydration. The maximum increase in sample strength on 28-day when the curing time and admixture amounts were 450.34% and 176.91%.
(2) The C&D waste has a positive effect on both the compressive and flexural properties of the samples, with the largest increase in performance being 57.2%. A 20% C&D waste content demonstrates the best-improving effect.
(3) Polypropylene fiber brings a 150.31% increase in the flexural properties of the samples. However, the increase in compressive properties is not significant.
(4) Higher levels of sodium sulphate increase the mechanical properties of the cement soil by 59.61% and 69.96%, respectively. However, the 0.4% sodium sulphate fails to change the properties regularly, with a range of −14% to 32.59%.
(5) The influencing factors of each variable on CSS performance are ranked in descending order as: Portland cement, polypropylene fiber, C&D waste, sodium sulfate. The mixture design of 30% cement, 20% C&D waste, 4% fiber and 0.8% is considered as the best-performed combination.
(6) BPNN and RF acquired the most accurate prediction for UCS and FS, respectively. Baseline models generally are inferior to Machine Learning models with hyperparameters in mechanical strength prediction.

The research output from this article could lead to a wider application of CSS as an engineering material. Moreover, the concluded enhancement can be treated as a baseline model. Future research can extend the experiments to explore other properties such as slump, or to consider alternative aggregate ratios. Meanwhile, RF and BPNN can be employed to predict whether the designed proportion will achieve the mechanical strength requirements or to optimize the proportioning for a given strength.

## Figures and Tables

**Table 1:** Physical and mechanical properties of soil sample.

Soil Properties	Value
Specific gravity	2.69
Liquid limit (%)	38.87
Plastic limit (%)	21.55
Plasticity index	17.32
Maximum dry unit weight (kN/m^3^)	1.51
Optimum moisture content (%)	25.37

**Table 2:** Physical and mechanical properties of polypropylene fiber.

Polypropylene Fiber Properties	Value
Diameter (μm)	10
Cut length (mm)	10
Density (g/cm^3^)	0.91
Tensile strength (MPa)	486
Stretching limit (%)	15
Acid resistance	Excellent
Alkali resistance	Excellent

**Table 3:** Evaluation of 5 ML models on UCS test group.

Evaluation Index	Model				
	LR	MLR	KNN	BPNN	RF
*RMSE* (MPa)	0.3694	0.2014	0.3242	0.1727	0.0280
R	0.9598	0.9462	0.8599	0.9594	0.9685

**Table 4:** Evaluation of 5 ML models on FS test group.

Evaluation Index	Model				
	LR	MLR	KNN	BPNN	RF
*RMSE* (MPa)	0.2386	0.1677	0.2498	0.1347	0.1583
R	0.9341	0.9107	0.7678	0.9446	0.9262

## Data Availability

The data presented in this study are openly available.

## Appendix A

**Table A1:** Mixing proportion for all variable combinations.

ID	UCS (MPa)			FS (MPa)		
	7-Day	14-Day	28-Day	7-Day	14-Day	28-Day
**Control 1**	0.0876	0.2503	0.3408	0.1242	0.1403	0.1571
**Control 2**	0.4820	0.5676	0.7560	0.1451	0.2375	0.3003
**Control 3**	1.2340	1.7832	1.9160	0.3891	0.5912	0.8316
**CWFS-1112**	0.4612	0.3225	0.3783	0.1645	0.1537	0.1943
**CWFS-1114**	0.4476	0.3439	0.4128	0.1544	0.1481	0.1904
**CWFS-1118**	0.5304	0.4448	0.5220	0.1684	0.1918	0.2886
**CWFS-1122**	0.4212	0.3862	0.5092	0.2006	0.2064	0.3543
**CWFS-1124**	0.4128	0.4212	0.5544	0.2584	0.3167	0.3715
**CWFS-1128**	0.4024	0.4236	0.5544	0.1904	0.2996	0.3488
**CWFS-1142**	0.4212	0.3225	0.4320	0.2519	0.2890	0.3356
**CWFS-1144**	0.4448	0.4236	0.5728	0.3581	0.3670	0.5117
**CWFS-1148**	0.4984	0.4876	0.6208	0.3692	0.3423	0.4967
**CWFS-1212**	0.3676	0.4104	0.5116	0.1643	0.1817	0.2264
**CWFS-1214**	0.4076	0.3811	0.5116	0.1700	0.1581	0.2094
**CWFS-1218**	0.4448	0.4636	0.6316	0.1832	0.2064	0.2761
**CWFS-1222**	0.3648	0.3462	0.4556	0.1589	0.1567	0.1893
**CWFS-1224**	0.3488	0.3597	0.4664	0.2232	0.2314	0.2285
**CWFS-1228**	0.5144	0.5464	0.7272	0.2398	0.3235	0.3210
**CWFS-1242**	0.4848	0.4664	0.5892	0.3698	0.4548	0.4739
**CWFS-1244**	0.3488	0.4716	0.5568	0.3774	0.3058	0.4289
**CWFS-1248**	0.4048	0.5676	0.6980	0.3274	0.4652	0.5195
**CWFS-1312**	0.3860	0.4392	0.5836	0.1674	0.1772	0.2363
**CWFS-1314**	0.3352	0.4156	0.5436	0.1517	0.1904	0.1998
**CWFS-1318**	0.3116	0.3676	0.5276	0.1195	0.1615	0.2421
**CWFS-1322**	0.2900	0.3890	0.5196	0.2296	0.3000	0.2977
**CWFS-1324**	0.2796	0.3304	0.4984	0.1799	0.3218	0.3270
**CWFS-1328**	0.3940	0.5220	0.6556	0.2710	0.2516	0.3739
**CWFS-1342**	0.3888	0.4368	0.5596	0.3164	0.4713	0.3691
**CWFS-1344**	0.3752	0.4528	0.6020	0.3032	0.4713	0.5394
**CWFS-1348**	0.4904	0.5436	0.8048	0.4861	0.5032	0.4442
**CWFS-2112**	0.6100	0.6848	0.9860	0.2753	0.3355	0.3807
**CWFS-2114**	0.6476	0.8236	1.0316	0.2911	0.3504	0.3821
**CWFS-2118**	0.7728	0.8500	1.1804	0.3175	0.4010	0.4440
**CWFS-2122**	0.7700	0.8796	1.2392	0.3898	0.4295	0.4914
**CWFS-2124**	0.8024	0.8528	1.1512	0.4256	0.4452	0.6076
**CWFS-2128**	0.8472	0.9888	1.2312	0.8139	0.7081	0.6401
**CWFS-2142**	0.6500	0.6208	1.0524	0.5446	0.7073	0.7116
**CWFS-2144**	0.7196	0.7808	1.0552	0.6068	0.6676	0.6396
**CWFS-2148**	0.7596	0.8528	1.0820	0.5692	0.6123	0.8199
**CWFS-2212**	0.5916	0.5856	0.9380	0.2905	0.2862	0.3238
**CWFS-2214**	0.5464	0.6528	0.8872	0.2567	0.3296	0.3459
**CWFS-2218**	0.7648	0.7300	1.1596	0.3668	0.4259	0.4089
**CWFS-2222**	0.6100	0.7436	0.9916	0.4029	0.5192	0.4870
**CWFS-2224**	0.5596	0.6820	0.8528	0.3065	0.3741	0.4079
**CWFS-2228**	0.7516	0.8100	1.0820	0.4071	0.3687	0.5402
**CWFS-2242**	0.6584	0.7516	0.9008	0.5067	0.5630	0.8492
**CWFS-2244**	0.8180	0.8100	1.1432	0.6622	0.7713	0.7164
**CWFS-2248**	0.7156	0.8436	1.1196	0.5821	0.8092	0.8442
**CWFS-2312**	0.7516	0.8552	1.0820	0.3391	0.4377	0.5259
**CWFS-2314**	0.6580	0.9140	1.1404	0.2974	0.4101	0.4568
**CWFS-2318**	0.7640	0.9702	1.2020	0.2985	0.3597	0.4502
**CWFS-2322**	0.7408	0.8553	1.0768	0.2919	0.3759	0.4609
**CWFS-2324**	0.8368	0.8846	1.2128	0.4333	0.4249	0.5002
**CWFS-2328**	0.9516	0.9931	1.3404	0.4617	0.5216	0.5790
**CWFS-2342**	0.8648	1.0981	1.4608	0.7216	0.7460	0.7958
**CWFS-2344**	0.8980	1.0153	1.5008	0.7840	0.7140	0.7119
**CWFS-2348**	0.9272	1.1304	1.4364	0.7981	0.8484	1.2859
**CWFS-3112**	1.1968	1.4952	2.0340	0.3985	0.5504	0.6006
**CWFS-3114**	1.0848	1.4444	1.8388	0.3893	0.5857	0.6095
**CWFS-3118**	1.5244	2.1324	2.5880	0.5919	0.8247	1.0207
**CWFS-3122**	1.1032	1.4900	1.8232	0.4669	0.6060	0.7354
**CWFS-3124**	1.4152	1.7780	2.2816	0.6744	0.8850	0.8449
**CWFS-3128**	1.3856	1.8444	2.2148	0.6531	0.9415	0.9202
**CWFS-3142**	1.4180	1.7752	2.2256	1.0989	1.1942	1.6690
**CWFS-3144**	1.5164	1.7404	2.1032	1.1547	1.4189	1.4320
**CWFS-3148**	1.3085	1.9538	2.4336	1.2395	1.3497	1.3127
**CWFS-3212**	1.3140	1.6258	2.0976	0.5497	0.6575	0.7450
**CWFS-3214**	1.3647	1.5537	2.0656	0.6025	0.6948	0.8935
**CWFS-3218**	1.2685	1.4820	2.0148	0.4875	0.4820	0.7792
**CWFS-3222**	1.3192	1.7296	2.0284	0.6980	0.8248	0.8717
**CWFS-3224**	1.2926	1.7164	2.1592	0.7482	0.8778	0.9954
**CWFS-3228**	1.3778	1.7780	1.9804	0.6061	0.7348	0.8046
**CWFS-3242**	1.4253	1.7324	2.1456	1.1684	1.6124	2.0286
**CWFS-3244**	1.3940	1.7216	2.1376	0.8412	0.8754	1.2671
**CWFS-3248**	1.5592	1.8868	2.3720	1.0135	1.4556	1.6752
**CWFS-3312**	1.2182	1.7564	2.1592	1.2259	0.7466	0.8835
**CWFS-3314**	1.3085	1.6284	2.3084	0.5386	0.7910	0.9581
**CWFS-3318**	1.0819	1.4632	1.8712	0.4022	0.5725	0.6951
**CWFS-3322**	1.1595	1.1056	1.7780	0.6079	0.6103	0.8183
**CWFS-3324**	1.0448	1.4128	1.5808	0.5543	0.5529	0.7040
**CWFS-3328**	1.4020	1.4100	1.9072	0.4953	0.6974	0.7057
**CWFS-3342**	1.5276	1.6736	1.9560	0.8984	1.2113	1.4504
**CWFS-3344**	1.2368	1.9432	1.7964	1.2807	1.2696	1.3083
**CWFS-3348**	1.9296	2.2656	2.6572	1.2370	1.1690	1.5334
